# Cross-cultural Validity and Reliability of the Recalled Parental Rearing Behavior Questionnaire

**Published:** 2018-12

**Authors:** Seongmi MOON, Dong Hee KIM, Yu-Mi IM, Sunhee LEE

**Affiliations:** 1. Dept. of Nursing, College of Medicine, University of Ulsan, Ulsan, Korea; 2. Dept. of Nursing, College of Nursing, Sungshin University, Seoul, Korea; 3. Dept. of Nursing, Seoul Women’s College of Nursing, Seoul, Korea; 4. Dept. of Nursing, College of Nursing, The Catholic University of Korea, Seoul, Korea

## Dear Editor-in-Chief

Parental style is known to affect childhood development, self-esteem, and school adjustment, and children with chronic disease have difficulty reaching these developmental milestones and adjusting to the school environment because of parental overprotection (1, 2). A scale to quantitatively measure parenting style is needed to research effective parenting style. The purpose of this study was to test the validity and reliability of the Korean version of the recalled parental rearing behavior (Fragebogen zum erinnerten elterlichen Erziehungsverhalten; FEE) questionnaire, developed in Germany.

The FEE is composed of the three factors emotional warmth, rejection/punishment, and control/overprotection and each factor has 8 items (3). The FEE is answered separately for susunn@1229 paternal and maternal rearing behaviors.

After author approval for using the FEE on Korean subjects, a Korean-German bilingual translator translated the FEE from German to Korean (FEE-K).

Then, another two Korean-German bilingual translators back-translated the Korean FEE to German and compared it with the original. A convenience sample of 272 adolescents aged 12 to 19 yr with congenital heart disease, leukemia, or solid carcinoma was selected at three university-affiliated hospitals from July 22 to August 23, 2013. Subjects were in follow-up after treatment. Overall, 260 answered questions regarding the rearing attitudes of their father, and 259 answered questions focused on their mother. Confirmatory factor analysis was performed with data on both paternal and maternal rearing behaviors to verify FEEK validity. The ratio of χ^2^ to degrees of freedom, goodness of fit index (GFI), adjusted goodness of fit index (AGFI), Tucker-Lewis index (TLI), comparative fit index (CFI), and root mean square error of approximation (RMSEA) were calculated to verify the fitness of the FEE-K structure. Factor loading, average variance extracted (AVE), and composite reliability (CR) were calculated to verify convergent validity of the FEE-K. Moreover, we compared AVE values to the squares of the correlation coefficients between factors to verify the discriminant validity of the FEE-K. To verify the reliability of the FEE-K, Cronbach’s alpha and average inter-item correlations were calculated.

The Korean version of the FEE was composed of three factors: emotional warmth, control/overprotection, and rejection/punishment. The indices for CFA model fit in the Korean version for three factors and 24 items needed improvement. To improve the model fit, we extracted the items with loading less than 0.4 and the items considered duplicated in terms of meaning. The final questionnaire included 16 items for fathers and 13 items for mothers. RMSEAs were below 0.8, showing reasonable fit. The AVEs of emotional warmth factor and rejection/punishment factor were higher than 0.500 in both fathers and mothers, indicating convergent validity. However, the AVE of the control/overprotection factor was 0.354 in fathers and 0.426 in mothers. The CRs ranged from 0.873 to 0.920 for both emotional warmth and rejection/punishment factors; however, they were less than 0.700 for the control/overprotection factor, implying that the internal consistency of the construct was not obtained. Therefore, we could not demonstrate the convergent validity of FEE-K.

The control/overprotection factor was not clearly discriminated, showing that the AVE of the control/overprotection factor was 0.354 in fathers and 0.426 in mothers, smaller than the square of the correlation coefficient between the control/overprotection factor and rejection/punishment factor, which was 0.635 in fathers and 0.587 in mothers (Table 1).

**Table 1:** Coefficients of determination among factors and average variance extracted (AVE)

***Factor***	***Father***			***Mother***		
	1	2	3	1	2	3
1. Emotional warmth	.521^*^			.565^*^		
2. Control/Overprotection	.004	.354^*^		.132	.426^*^	
3. Rejection/Punishment	.029	.635	.668^*^	.091	.587	687^*^

The Cronbach’s alpha of FEE-K in fathers was 0.733, and that in mothers was 0.700. The values of Cronbach’s alpha were higher than 0.700 for the emotional warmth factor and rejection/punishment factor in both fathers and mothers; however, for the control/overprotection factor, Cronbach’s alpha was 0.500 in fathers and 0.480 in mothers. The average inter-item correlations were all acceptable, ranging from 0.259 to 0.511.

Our findings indicated that the perceived parenting styles of Korean children and adolescents with chronic disease are different from those of Western children and adolescents. Especially, control/overprotection factor did not show convergent and discriminant validity. In Korea, parents should make decisions for their child, and children should follow the decision of their parents. Accordingly, Korean children tend to recognize that they have to change their own behavior to prevent or resolve conflict with their parents (4). Especially, adolescents with chronic disease have a high level of dependence relative to healthy adolescents because of their illness and feel guilty that their parents suffer due to their chronic illness (5). Therefore, participants have difficulty expressing their emotion and need when parental conflicts occur and take their parent’s needs for granted. Participants tend to perceive parental control/overprotection factors as love and have difficulty recognizing whether or not parental behavior is overprotective.

Reliability coefficients of the control/overprotection factor were also lower than 0.700, indicating that Korean family culture is different from Western family culture, and future research about how parenting style affects children and adolescents with chronic disease in Korean family culture is necessary.
